# Myocardial dysfunction in patients with type 2 diabetes mellitus: role of endothelial progenitor cells and oxidative stress

**DOI:** 10.1186/1475-2840-11-147

**Published:** 2012-12-05

**Authors:** Chun Ting Zhao, Mei Wang, Chung Wah Siu, Ying Long Hou, Tian Wang, Hung Fat Tse, Kai Hang Yiu

**Affiliations:** 1 Shan Dong University School of Medicine, Shandong, China; 2 Division of Cardiology, Department of Medicine, the University of Hong Kong, Queen Mary Hospital, Rm 1929b, Block K, Hong Kong, China; 3 Division of Cardiology, Traffic Hospital in Shan Dong Province, Shandong, China; 4 Research Centre of Heart, Brain, Hormone and Healthy Aging, Li Ka Shing Faculty of Medicine, the University of Hong Kong, Hong Kong, China

**Keywords:** Type 2 diabetes mellitus, Myocardial injury, Endothelial progenitor cells

## Abstract

**Background:**

Endothelial progenitor cells (EPCs) are responsible for angiogenesis and maintenance of microvascular integrity, the number of EPCs is correlated with oxidative stress. Their relation to myocardial dysfunction in patients with type 2 diabetes mellitus (T2DM) is nonetheless unknown.

**Methods:**

Eighty-seven patients with T2DM and no history of coronary artery disease were recruited. Transthoracic echocardiography and detailed evaluation of left ventricular (LV) systolic function by 2-dimensional (2D) speckle tracking derived strain analysis in 3 orthogonal directions was performed. Four subpopulations of EPCs, including CD34+, CD133+, CD34+/kinase insert domain-containing receptor (KDR) + and CD133+/KDR + EPCs, were measured by flow cytometry. Oxidative stress was assessed by superoxide dismutase (SOD).

**Results:**

The mean age of the patients was 62 ± 9 years and 39.6% were male. Those with an impaired longitudinal strain had a lower number of CD34+ EPCs (2.82 ± 1.87% vs. 3.74 ± 2.12%, P < 0.05) than those with preserved longitudinal strain. When compared with those with preserved circumferential strain, patients with an impaired circumferential strain had a lower number of CD34+ EPCs (2.63 ± 1.80% vs. 3.87 ± 2.10%, P < 0.01) and SOD level (0.13 ± 0.06U/ml vs. 0.20 ± 0.08U/ml, P < 0.01). Patients with an impaired radial strain nonetheless had a lower number of CD34+ EPCs (2.62 ± 2.08% vs. 3.69 ± 1.99%, P < 0.05). Multivariate analysis demonstrated that only impaired global circumferential strain remained significantly associated with CD34 + EPCs and SOD.

**Conclusions:**

LV global circumferential strain was independently associated with number of CD34+ EPCs and SOD. These findings suggest that myocardial dysfunction in patients with T2DM is related to depletion of EPCs and increased oxidative stress.

## Introduction

Type 2 diabetes mellitus (T2DM) is associated with myocardial dysfunction, independent of underlying coronary artery disease. Patients with T2DM have a 2–5 fold higher risk of developing heart failure than those without [1,2]. Although the pathology of myocardial dysfunction in these patients is unclear, it is likely multifactorial and includes increased oxidative stress, [3] microangiopathy, [4] and altered myocardial metabolism and structure with fibrosis [5].

Increasing evidence is emerging to support the role of endothelial progenitor cells(EPCs), [6] a subpopulation of mononuclear cells that possess the ability for vascular repair and neovascularization, in microangiopathy and myocardial dysfunction in patients with T2DM [7,8]. In addition, EPCs have been shown to be closely related with oxidative stress, that also contributes to myocardial dysfunction in these patients. Recent studies have demonstrated that 2-dimensional (2D) speckle tracking derived strain analysis is a sensitive method to detect systolic dysfunction in T2DM patients with an apparently normal left ventricular (LV) ejection fraction [9,10]. Nevertheless the association of EPCs and oxidative stress with LV myocardial function in patients with T2DM has not been evaluated. The aim of the present study is to determine the relationship of EPCs and oxidative stress, as determined by superoxide dismutase (SOD), to myocardial function measured by 2D speckle tracking LV strain.

## Method

### Study population

A total of 110 consecutive patients with T2DM as defined by World Health Organization criteria and no coronary artery disease were recruited at Queen Mary Hospital from January 2008 to January 2010. Patients were excluded if they had a documented history or clinical symptoms and signs of macrovascular disease including myocardial infarction, coronary artery disease, stroke or peripheral vascular disease. Patients with dilated cardiomyopathy, New York Heart Association class III/IV heart failure, significant renal dysfunction with creatinine level > 220 umol/L, liver failure or clinical/biochemical evidence of concomitant inflammatory disease or patients who declined to participate were also excluded. As a result, 87 subjects were eligible for this study. The current study is in compliance with the Helsinki Declaration and has been approved by the Hong Kong West Cluster Ethics Committee. All patients had a written informed consent.

### Study design

Baseline demographic data and cardiovascular medications were recorded in all subjects. Hypertension was defined as resting systolic or diastolic blood pressure ≥140 /90 mmHg on two occasions or the prescription of anti-hypertensive medication. Hypercholesterolemia was defined as fasting total plasma cholesterol level ≥4.9 mmol/L or the prescription of lipid-lowering medication [11]. Smoking status was recorded as ever-smoker (past or current) or non-smoker. Body height, weight and blood pressure were measured as previously described [12]. Body-mass index (BMI) was calculated as kg/m^2^. Fasting blood samples were obtained to measure serum total cholesterol, triglyceride, low-density lipoprotein-cholesterol (LDL-C), high-density lipoprotein-cholesterol (HDL-C), glucose, HbA1c, creatinine, and high sensitivity C-reactive protein (hs-CRP).

After mixing, samples were centrifuged at 3000 rpm for 20 minutes and the supernatants frozen at −80°C until assay. Oxidative stress was measured as superoxide dismutase (SOD), a potent antioxidant enzyme [13].

### Flow cytometry

Four subpopulations of EPCs, including CD34+, CD133+, CD34+/kinase insert domain-containing receptor (KDR) + and CD133+/KDR + EPCs, were measured by flow cytometry. Fluorescence-activated cell analysis was performed to determine the number of EPCs as described previously [14]. Briefly, 100 μl of peripheral blood was incubated with a phycoerythrin-conjugated monoclonal antibody against human KDR (Sigma, St Louis, MO, USA), followed by a fluorescein isothiocyanate (FITC)-conjugated CD34 and CD133 antibody (Beckman Coulter, Fullerton, CA, USA). FITC-labelled anti-human CD45 antibody was used for differential gating during flow analysis. FITC-labelled IgG1a (Beckman Coulter) and phycoerythrin-labelled IgG2b (Becton Dickinson, Franklin Lakes, NJ, USA) served as the isotypic control for colour compensation. Analysis was performed with an automated fluorescence-activated cell counter (Elite; Beckman Coulter) in which 1 000 000 events were counted. The absolute number of cells expressing CD34+, CD133+, CD34/KDR + CD133/KDR + per 1 000 000 events in the lymphocyte gate was calculated. The percentages of all the measured components were derived from the absolute cell count divided by the lymphocyte count [15].

### Echocardiography

Transthoracic echocardiography was performed in all patients using a commercially available system (Vingmed Vivid 7, General Electric Vingmed Ultrasound, Milwaukee, USA). A 3.5-MHz transducer was used to obtain images that were digitally stored in cine-loop format (5 cardiac cycles). Measurements were performed offline using EchoPAC version 108.1.5 (General Electric – Vingmed, Horten, Norway). The inter-ventricular septum thickness, posterior wall thickness, and LV dimensions were measured in M-modal according to the current recommendation. LV volume and ejection fraction was determined from apical four and two-chamber views using the modified Simpson’s biplane method of discs. Evaluation of LV diastolic function was based on the pulsed-wave Doppler of mitral valve inflow. Peak velocity in early diastole (E-wave) and late diastole (A-wave) was measured and the E/A ratio calculated. Pulsed wave tissue Doppler imaging was used to measure the early diastolic velocity (E’) with the sample volume placed at lateral annulus. In addition, E/E’ ratio was calculated as an estimation of LV filling pressure [16]. LV diastolic dysfunction was therefore classified as previously described [17].

### Two-dimensional speckle tracking strain analysis

Two-dimensional speckle tracking strain analysis allows detailed assessment of LV myocardial deformation by tracking natural acoustic markers (speckles) in a frame-to-frame basis within the cardiac cycle. LV deformation can be evaluated in three orthogonal directions as longitudinal, circumferential and radial strains [18].

Longitudinal strain, assessing the shortening/lengthening of the myocardial wall, was measured from the 3 apical views: 2-chamber view (comprising anterior and inferior walls), 4-chamber view (posteroseptal and lateral walls) and long axis view (anteroseptal and posterior walls). Each wall was subsequently divided into 3 levels (basal, mid and apical) and a total of 18 segmental strain curves were obtained. Global longitudinal strain was calculated as the mean of the peak systolic strain value of the 18 segments.

From LV mid-ventricular short-axis view, both circumferential strain (evaluating myocardial shortening/lengthening along LV curvature) and radial strain (evaluating myocardial thickening/thinning) were measured. The global value of circumferential and radial strains were derived from the average peak systolic strain value of 6 segments. Global longitudinal and circumferential strains are expressed as negative values, and a lower strain is represented by less negative values. Global radial strain is expressed as a positive value: a lower value indicates lower strain.

Impairment of the 3-orthogonal directional global strains was defined as mean ± 2 standard deviations according to the results of a recent study that assessed global strains in healthy Asian subjects using the same vendor machine as the current study [19]. Thus in this study impaired global longitudinal strain is defined as > −17.1%; impaired global circumferential strain > −17.0% and; impaired radial strain <29.4%.

The interobserver and intraobserver variability for longitudinal, radial, and circumferential strains were 6.5% and 2.6%, 10.4% and 7.6%, 4.9% and 2.4%, respectively.

### Statistical analysis

All continuous variables and categorical variables are expressed as mean ± standard deviation and frequencies or proportions, respectively. Continuous demographic variables were compared using the Mann–Whitney *U* test and categorical demographic variables were compared using Pearson Chi-square test or the Fisher’s exact test if at least one cell had an expected cell count below five. Correlation coefficients were performed in order to assess the association of circulating EPCs and oxidative stress with myocardial function in T2DM patients. Multivariate analyses were performed to detect the predictors for abnormal myocardial function. To avoid multi-collinearity, multi-directional strains were entered individually into the model. All statistical analyses were performed using the statistical package SPSS for windows (Version 18.0, SPSS, Chicago, USA). All P values reported are 2-sided for consistency. A P value *<0.05* was considered statistically significant.

## Results

### Baseline characteristics

The baseline characteristics of all patients are shown in Table 1. All patients had normal LV dimension and function including LV end diastolic volume (mean 80 ± 19 ml), LV end systolic volume (30 ± 12 ml) and LV ejection fraction (64 ± 7%). Diastolic dysfunction was noted in 69 patients (79%) and the mean E/E’ ratio was 9.9 ± 2.6. The mean global longitudinal, circumferential and radial strains were 17.6 ± 2.5%, 17.7 ± 2.2% and 33.7 ± 11.0%, respectively. According to the pre-defined cut-off values, 39% of patients had impaired global longitudinal strain; 39% had impaired radial strain; and 30% had impaired radial strain.

**Table 1:** Clinical demographics in patients with and without impaired strains

**Variables**	**All patients (n = 87)**	**Longitudinal strain (cutoff −17.1%)**		**Circumferential strain (cutoff −17.0%)**		**Radial strain (cutoff 29.4%)**	
		**Preserved (n = 53)**	**Impaired (n = 34)**	**Preserved (n = 53)**	**Impaired (n = 34)**	**Preserved (n = 62)**	**Impaired (n = 25)**
Age, years	61.8 ± 9.3	60.5 ± 9.7	63.9 ± 8.2	61.4 ± 10.1	62.5 ± 7.9	61.3 ± 9.7	62.9 ± 8.3
Male gender, % (n)	39.6(36)	42.9(24)	34.3(12)	33.9(19)	48.6(17)	59.7(37)	64.0(16)
BMI, kg/m^2^	25.7 ± 4.3	^*^25.01 ± 4.2	26.9 ± 4.2	25.3 ± 4.0	26.3 ± 4.7	25.2 ± 4.4	26.9 ± 4.2
SBP, mmHg	137 ± 16	137 ± 16	138 ± 16	138 ± 15	138 ± 17	136 ± 16	141 ± 16
DBP, mmHg	78 ± 8	78 ± 8	78 ± 8	77 ± 9	78 ± 7	77 ± 8	79 ± 8
Duration of disease, years	9.9 ± 7.7	8.9 ± 8.0	11.8 ± 7.0	8.9 ± 6.9	11.7 ± 8.9	10.4 ± 8.2	9.0 ± 7.2
Insulin therapy, % (n)	19.8(18)	^*^37.1(13)	8.9(5)	19.6(11)	20.0(7)	17.7(11)	24.0(6)
Smoker, % (n)	17.6(16)	17.9(10)	17.1(6)	16.1(9)	20.0(7)	14.5(9)	20.0(5)
Hypertension, % (n)	60.4(55)	57.1(32)	65.7(23)	57.1(32)	65.7(23)	61.3(38)	52.0(13)
Total cholesterol, mmol/L	4.9 ± 0.8	5.02 ± 0.90	4.83 ± 0.73	5.01 ± 0.85	4.84 ± 0.82	4.97 ± 0.86	4.95 ± 0.78
Triglycerides, mmol/L	1.4 ± 0.8	1.36 ± 0.80	1.42 ± 0.75	1.43 ± 0.79	1.31 ± 0.77	1.32 ± 0.77	1.53 ± 0.81
High density lipoprotein, mmol/L	1.4 ± 0.4	1.39 ± 0.37	1.44 ± 0.45	1.40 ± 0.36	1.42 ± 0.45	1.42 ± 0.45	1.40 ± 0.24
Low density lipoprotein, mmol/L	2.9 ± 0.7	3.01 ± 0.75	2.72 ± 0.62	2.95 ± 0.70	2.83 ± 0.74	2.93 ± 0.71	2.86 ± 0.72
Fasting glucose, mmol/L	7.57 ± 2.07	7.19 ± 1.78	8.11 ± 2.34	7.49 ± 2.31	7.68 ± 1.68	7.68 ± 2.19	7.52 ± 1.70
Creatinine, umol/L	77.1 ± 25.1	76.8 ± 27.58	77.6 ± 21.1	77.3 ± 28.5	76.8 ± 19.0	74.1 ± 15.9	77.1 ± 23.6
hs-CRP, mg/L	1.49 ± 2.18	1.36 ± 1.75	1.72 ± 2.80	1.65 ± 2.71	1.27 ± 0.98	1.33 ± 1.68	1.90 ± 3.09
SOD, U/ml	0.17 ± 0.08	0.18 ± 0.09	0.15 ± 0.06	^**^0.20 ± 0.08	0.13 ± 0.06	0.17 ± 0.09	0.16 ± 0.08
HbA1c, %	7.72 ± 1.24	^*^7.51 ± 1.22	8.07 ± 1.20	7.70 ± 1.21	7.75 ± 1.30	7.83 ± 1.32	7.54 ± 1.00
CD34+ EPCs, %	3.38 ± 2.07	^*^3.74 ± 2.12	2.82 ± 1.87	^**^3.87 ± 2.10	2.63 ± 1.80	*3.69 ± 1.99	2.62 ± 2.08
CD133+ EPCs, %	0.44 ± 0.45	0.44 ± 0.47	0.45 ± 0.43	0.50 ± 0.49	0.35 ± 0.38	0.46 ± 0.47	0.41 ± 0.42
CD34+/KDR + EPCs, %	0.85 ± 0.69	0.89 ± 0.57	0.78 ± 0.85	0.92 ± 0.78	0.74 ± 0.52	0.91 ± 0.75	0.70 ± 0.49
CD133+/KDR + EPCs, %	0.25 ± 0.28	0.25 ± 0.30	0.25 ± 0.28	0.28 ± 0.32	0.21 ± 0.20	0.28 ± 0.32	0.19 ± 0.14

* P < 0.05; ** P < 0.01.

Abbreviations: *BMI* = body mass index; *EPCs* = endothelial progenitor cells; *DBP* = diastolic blood pressure; *hs-CRP* = High sensitivity C-reactive protein; *SBP* = systolic blood pressure; *SOD* = superoxide dismutase.

### Clinical demographics in patients with and without impaired strains

The clinical demographics of patients with and without impaired LV strain are shown in Table 1. Patients with an impaired longitudinal strain had a higher BMI, HbA1c level and a lower number of CD34+ EPCs than those with preserved longitudinal strain. When compared with those with preserved circumferential strain, patients with an impaired circumferential strain had a lower CD34+ EPCs and SOD level. Patients with an impaired radial strain nonetheless had a lower number of CD34+ EPCs compared with those with preserved radial strain.

### Correlation of global strains with clinical demographics

The correlation between the 3 orthogonal directional strains and clinical demographics is shown in Table 2. Global longitudinal and radial strains were both negatively correlated with BMI. Global circumferential and radial strains were correlated with CD34+ EPCs. Only circumferential strain was negatively correlated with serum level of SOD. In addition, level of CD34+ EPCs was significantly correlated with serum level of SOD (R = 0.37, *P < 0.01*). Nonetheless CD133+ (R = 0.13, *P = 0.30*), CD34+/KDR + (R = 0.09, *P = 0.50*) and CD133+/KDR + EPCs (R = 0.11, *P = 0.41*) showed no such correlation.

**Table 2:** Correlation of the cardiovascular risk factors and global strains

**Variables**	**Longitudinal strain**		**Circumferential strain**		**Radial strain**	
	**R**	**P value**	**R**	**P value**	**R**	**P value**
Age	0.19	0.08	−0.01	0.92	−0.15	0.17
BMI	0.22	^*^0.04	0.19	0.09	−0.32	^*^0.003
SBP	0.08	0.44	0.08	0.50	−0.14	0.21
DBP	0.04	0.71	0.11	0.33	−0.12	0.30
Duration of disease	0.15	0.18	0.05	0.66	0.03	0.82
Total cholesterol	−0.04	0.74	−0.08	0.48	−0.05	0.67
Triglycerides	0.13	0.23	−0.05	0.67	−0.15	0.18
High density lipoprotein	−0.03	0.81	0.03	0.76	0.03	0.78
Low density lipoprotein	−0.10	0.33	−0.09	0.42	0.07	0.95
Fasting glucose	0.10	0.38	0.07	0.57	0.01	0.92
Creatinine	0.08	0.48	0.16	0.14	−0.10	0.39
hs-CRP	0.05	0.72	−0.01	0.92	−0.22	0.09
SOD	−0.23	0.07	−0.32	^*^0.01	0.11	0.39
HbA1c	0.07	0.50	−0.12	0.29	0.07	0.53
CD34+ ECPs	−0.19	0.08	−0.28	^*^0.01	0.21	^*^0.049
CD133+ EPCs	−0.02	0.86	−0.08	0.47	0.02	0.86
CD34+/KDR + EPCs	−0.01	0.96	−0.21	0.05	0.18	0.10
CD133+/KDR + EPCs	−0.07	0.54	−0.16	0.14	−0.02	0.87

Abbreviations: *BMI* = body mass index; *EPCs* = endothelial progenitor cells; *DBP* = diastolic blood pressure; *hs-CRP* = High sensitivity C-reactive protein; *SBP* = systolic blood pressure; *SOD* = superoxide dismutase.

### Association of endothelial progenitor cells and superoxide dismutase with global strains

Univariate analysis revealed that impaired circumferential and radial strains were associated with CD34+ EPC level and impaired global longitudinal, circumferential and radial strains were related to SOD (Table 3). In order to evaluate the relation of impaired global strains with CD34+ EPCs and SOD, multivariate adjustment for age, gender, BMI, smoking history, hypertension, hypercholesterolaemia and HbA1c was performed. The results demonstrated that impaired global circumferential and radial strains were independently associated with CD34+ EPCs, whereas only impaired global circumferential strain was independently associated with SOD. When CD34+ EPCs and SOD were evaluated together, impaired global circumferential strain was the only LV strain that remained significantly associated with both CD34 + EPCs (odd ratio [OR]. = 0.64, Confidence interval [CI] = 0.43 - 0.95, P = 0.03) and SOD (OR = 0.001, CI = 0.001 – 0.23, P = 0.02).

**Table 3:** Unadjusted and adjusted effects of superoxide dismutase (SOD) and CD34+ endothelial progenitor cells (EPCs) on global strains

	**Longitudinal strain**		**Circumferential strain**		**Radial strain**	
	**OR (95%)**	**P value**	**OR (95%)**	**P value**	**OR (95%)**	**P value**
SOD						
Uadjusted	0.002(0.001,1.544)	<0.01	0.001(0.001, 0.009)	<0.01	0.02 (0.01, 17.22)	0.02
#Adjusted	0.001(0.000, 1.353)	0.060	0.002(0.001, 0.007)	<0.01	0.004 (0.001, 7.868)	0.15
CD34+ EPCs						
Uadjusted	0.81 (0.65, 1.02)	0.07	0.71 (0.55, 0.92)	<0.01	0.75 (0.57, 0.98)	0.03
#Adjusted	0.82 (0.64-1.05)	0.12	0.71 (0.53, 0.93)	0.01	0.74 (0.56, 0.98)	0.04
CD133+ EPCs						
Uadjusted	0.87 (0.35, 2.18)	0.76	0.44 (0.15, 1.32)	0.15	0.77 (0.26, 2.26)	0.77
#Adjusted	0.51 (0.18, 1.46)	0.21	0.38 (0.12, 1.20)	0.10	0.46 (0.14, 1.58)	0.22
CD34/KDR + EPCs						
Uadjusted	0.79 (0.41, 1.51)	0.47	0.64 (0.30, 1.35)	0.24	0.56 (0.23, 1.37)	0.21
#Adjusted	0.71 (0.36, 1.42)	0.33	0.66 (0.30, 1.49)	0.32	0.50 (0.19, 1.33)	0.17
CD133/KDR + EPCs						
Uadjusted	0.72 (0.17, 3.12)	0.66	0.34 (0.05, 2.17)	0.25	0.23 (0.02, 2.35)	0.21
#Adjusted	0.30 (0.06, 1.57)	0.15	0.26 (0.04, 1.79)	0.19	0.09 (0.01, 1.20)	0.07

Abbreviations: *BMI* = body mass index; *EPCs* = endothelial progenitor cells; *DBP* = diastolic blood pressure; *hs-CRP* = High sensitivity C-reactive protein; *SBP* = systolic blood pressure; *SOD* = superoxide dismutase.

#Adjusted for age, gender, BMI, smoking, hypertension, hypercholesterolaemia, hbA1c.

## Discussion

The present study demonstrated that at least 30% of T2DM patients, with apparently normal LV dimensions and ejection fraction, have impaired myocardial function as measured by 2D speckle tracking derived strain. Importantly, LV global circumferential strain was independently associated with depletion of CD34+ EPCs and increased oxidative stress measured by SOD.

### Endothelial progenitor cells and myocardial dysfunction

The pathogenesis of myocardial dysfunction is multifaceted, but includes activation of renin-angiotensin aldosterone system, [20] myocardial steatosis, [21] autonomic dysfunction [22] and increased myocardial fibrosis [23]. Microangiopathy nonetheless has been proposed to contribute to diabetic heart disease [24,25].

The presence of diabetes induces thickening of the capillary basement membrane and endothelial swelling of the myocardium [26,27]. These changes give rise to microangiopathy as evidenced by microaneurysms, interstitial fibrosis, and perfusion defect by radiological study and finally result in myocardial dysfunction [28]. Preserving the integrity of the microvasculature, which is partly maintained by circulating EPCs, may thus prevent the development of diabetic heart disease. In addition to endothelial regeneration and postnatal neovascularization in the ischemic region, the ability to differentiate into mature endothelial cells and be incorporated into new vessels enables EPCs to produce pro-angiogenic cytokines in a paracrine fashion to facilitate vascularization [29,30]. The clinical value of EPCs has been established in diabetic patients, and their depletion is associated with macrovascular disease such as increased arterial stiffness, [31] carotid intima media thickness, [32] brachial flow mediated dilatation [33] and peripheral artery disease [34,35]. Depletion of EPCs has also been shown to be predictive of future adverse cardiovascular events [36,37]. A study by Yoon and colleagues has shown that *DiI* labelled bone marrow derived EPCs contributed to myocardial microvasculature and function in rats with diabetic heart disease [4]. No studies have evaluated the role of EPCs with myocardial dysfunction in human subjects. The present study further demonstrated that EPCs in patients with T2DM and no atherosclerotic disease, were independently associated with myocardial dysfunction, measured by 2D speckle tracking strain. The current results thus suggest that depletion of EPCs contributes to the development of diabetic heart disease.

The present results indicate that CD34+ EPCs were most strongly correlated with myocardial dysfunction, compared with other subtypes of EPCs. Although the reason is uncertain, this could be partly explained by the ability of CD34+ EPCs to differentiate not only into hematopoietic stem cells, but also cardiomyocytes, smooth muscle and endothelial cells [38]. Previous studies have shown the CD34+ EPCs are best related with cardiovascular risk factors, metabolic syndrome [39] and long-term outcome compared with other subtypes of EPCs [36]. The multi-lineage property of CD34+ EPCs may thus provide a more comprehensive assessment of the pathophysiological development of diabetic heart disease.

### Oxidative stress and myocardial dysfunction

Oxidative stress, represented by an overproduction of reactive oxygen species, plays a role in all stages of diabetic heart disease, ranging from cardiac hypertrophy to myocardial fibrosis and dysfunction [40]. Animal studies have shown that suppression of oxidative stress with coenzyme A10 (an antioxidant in its reduced form, ubiquinol-10) [41] and 3′,4′-dihydroxyflavonol (a synthetic flavonol) [42], improves cardiac function and reduces myocyte hypertrophy and collagen deposition in diabetic rats. The present results further demonstrate the independent role of SOD in relation to myocardial dysfunction, thus highlighting the role of oxidative stress in the development of diabetic heart disease. In a study of 23 patients with T2DM, SOD has been proven to play a role in modulating EPC function under hyperglycemic conditions [43]. This finding is further confirmed by the results of this study, suggesting the close interplay between oxidative stress and EPCs that independently contributes to myocardial dysfunction.

### Clinical implications

The present study is the first to demonstrate that impaired myocardial function in patients with T2DM is independently associated with depletion of EPCs and increased oxidative stress. In animal studies, therapeutic interventions aimed at increasing EPCs and reducing oxidative stress improved myocardial function [4]. Future human studies should therefore aim to develop therapies that may reduce oxidative stress and/or recruit EPCs to prevent myocardial dysfunction in patients with T2DM.

### Limitations

A causal relationship between CD34+ EPCs, SOD and impaired myocardial strain could not be established because of the cross-sectional nature of the study. Further, although patients with T2DM were all clinically free of cardiovascular complications, the presence of asymptomatic coronary artery disease could not be excluded. The small study population did not allow additional analysis of other mechanisms that could potentially contribute to myocardial dysfunction. In addition, the reason for the correlation of CD34+ EPCs and SOD with only global circumferential strain, not longitudinal and radial strains, is uncertain. The LV myocardial fibre architecture has a typical orientation of myocardial strands that change from being oblique in the subepicardium, to circumferential in the middle, and longitudinal in the subendocardium [44]. Thus, whether depletion of CD34+ EPCs and oxidative stress preferentially affects the mid-wall, rather than the subendocardium in these patients, requires further evaluation.

## Conclusions

Patients with T2DM and no clinical evidence of macrovascular disease showed impaired myocardial strain detected by 2D speckle tracking derived strain analysis. Importantly, LV global circumferential strain was independently associated with both CD34+ EPCs and SOD. These findings suggest that myocardial dysfunction in patients with T2DM is related to depletion of EPCs and increased oxidative stress.

## Abbreviations

2D: 2-dimensional; BMI: Body-mass index; EPCs: Endothelial progenitor cells; HDL-C: High-density lipoprotein-cholesterol; hs-CRP: High sensitivity C-reactive protein; LDL-C: Low-density lipoprotein-cholesterol; LV: Left ventricular; SOD: Superoxide dismutase; T2DM: Type 2 diabetes mellitus.

## Competing interests

The authors declare that there is no competing interest associated with this manuscript.

## Authors’ contributions

CTZ coordinated analyses, interpreted results and wrote the manuscript. MW performed statistical analyses and revised the manuscript. CWS advised and supervised statistical analyses and revised the manuscript. YLH and TW were involved in the conception and design of the study, interpretation of the data, ongoing support and advice to the first author. KHY contributed to study design, data interpretation and manuscript revision. HFT designed and led the project and revised the manuscript. All authors have approved the final version of the manuscript.
